# Status of professional mental health help-seeking intention associated factors among medical students: a cross-sectional study in China

**DOI:** 10.3389/fpsyt.2024.1376170

**Published:** 2024-06-04

**Authors:** Lei Qiu, Kaixin Wangzhou, Yudan Liu, Jindong Ding, Hui Li, Jinhui Ma

**Affiliations:** ^1^ International School of Public Health, Hainan Medical University, Hainan, China; ^2^ School of Management, Hainan Medical University, Hainan, China; ^3^ School of Public Health, North Sichuan Medical College, Sichuan, China; ^4^ Research Unit for General Practice, Department of Public Health, University of Copenhagen, Copenhagen, Denmark; ^5^ Research Unit for General Practice, Department of Public Health, University of Southern Denmark, Odense, Denmark; ^6^ School of Health Services Management, Anhui Medical University, Hefei, China

**Keywords:** stigma, help-seeking intention, medical students, China, mental health

## Abstract

**Aim:**

Low professional help-seeking intention (PHSI) hinders effective treatment of mental illness. PHSI among Chinese students is still understudied and under-recognized. This study aimed to evaluate the status of PHSI and its associated risk factors among Chinese medical students.

****Method**s:**

A cross-sectional survey was conducted in Hainan province, South China, between January 1, 2021, and May 31, 2021. A total of 2182 medical students were recruited and surveyed via an anonymous structured questionnaire. Logistic regression analyses were performed to identify the factors associated with PHSI.

**Results:**

Among the 2182 medical students (mean age 21.0 years (SD = 3.70), 61.5% females), those with and without PHSI were 72.0% and 28.0%, and 16.4% with moderate to severe depression. Male students, those with a high level of depression stigma, serious family dysfunction, and heavy dependence on mobile phones were significantly less likely to seek professional mental health help, with odds ratios (ORs) of 1.5, 2.0, 2.1, and 1.7, respectively.

**Conclusion:**

A significant proportion of Chinese medical students demonstrate low PHSI, influenced by factors such as gender, depression stigma, family dysfunction, and mobile phone dependence. Future interventions aimed at increasing medical students’ PHSI should prioritize reducing depression stigma, mitigating reliance on mobile phone use, and enhancing family function to address these key barriers to seeking professional mental health support.

## Introduction

Mental health issues among university students are receiving increasing attention owing to the prevalence of these issues on campus (1). Actively seeking psychological help is a way to maintain health when suffering from depression and other mental diseases. Professional help-seeking intention (PHSI) is defined as an individual’s subjective likelihood of seeking help from a mental health professional. Most colleges and universities have also established mental health institutions to provide professional psychological counseling or services related to mental health. However, the prevalence of PHSI is low, and campus mental health services are underutilized.

Many studies have shown that when college students experience psychological distress or problems, only a small percentage seeks professional psychological assistance (2–4). When individuals encounter mental health problems, negative-seeking behavior is conducive to effectively solving psychological problems. It causes individuals to miss potential rehabilitation opportunities and aggravate their psychological problems (5). A better understanding of PHSI could inform the design of a comprehensive prevention strategy for medical students and help mental health workers to provide better campus psychological services.

According to studies, the long years of medical studies and the painful environments expose medical students to patient suffering and death. The demanding academic requirements of becoming a medical professional are also risk factors that cause stress and have a negative impact on student’s mental well-being (6). In addition to these academic pressures, individual factors such as personal or family backgrounds and individual characteristics also play a crucial role in the mental health of medical students. These factors can influence how students cope with stress, their likelihood of experiencing mental health issues, and their willingness to seek help (7). Furthermore, the prevalence of mental health issues such as stress, anxiety, and psychological distress among medical students is alarmingly high, underscoring the need for effective on-campus mental health services and interventions. These services are essential for early detection, support, and management of mental health concerns, contributing to the overall well-being of the student population (5, 6). Moreover, in the Chinese context, family dynamics and functionality significantly influence students’ mental health and their propensity to seek help. The traditional emphasis on family harmony and responsibility can impact individuals’ willingness to seek external assistance for mental health issues, often prioritizing family image and expectations over personal well-being. This cultural aspect underscores the importance of considering family function as a crucial variable in understanding PHSI among Chinese medical students.

Such a high prevalence of depression or depressive symptoms was reported in America (59.1%) (8), Saudi Arabia (58.4%) (9), Pakistan (52.4%) (10) and Korea (37.1%) (11). The overall prevalence of depressive symptoms among medical students varies greatly according to different studies. A meta-analysis found that the overall prevalence of depression or depressive symptoms among medical students was 27.2% (12). Given the high prevalence of depression and its significant impact, understanding the stigma associated with depression among medical students is crucial. Stigma can deter students from seeking the help they need, exacerbating the issue and hindering recovery. Addressing this stigma is therefore a key aspect of improving mental health outcomes for medical students (11). Depression in medical students can have long-term effects on individual emotional, social, physical, and cognitive development, leading to various negative outcomes (2). Therefore, students should be encouraged to seek professional psychological help when or possibly before the disease occurs or develops.

Professional psychological help-seeking is a way of coping with mental illnesses. Researchers define this type of assistance as seeking emotional support, instrumental support, advice, or information from professional psychologists or counselors to avoid or reduce stress or its consequences (13). There is considerable literature on socio-demographic factors associated with PHSI, including gender, grade, and family economic situation (14–18).

Furthermore, psycho-social factors like social support and depression stigma may be linked to PHSI (19). However, the impact of the above-mentioned factors on PHSI has been complex, with inconsistent findings from different studies. Although the research on PHSI and related behaviors has increased, PHSI among Chinese students remains understudied and under-recognized.

Stigma is a socially unacceptable flaw or mark caused by personal or physical characteristics (20). Individuals suffering from depression face social avoidance, discrimination, and lower self-efficacy and self-esteem due to stigma (21). Depression stigma includes personal depression stigma and perceived depression stigma (22). Personal depression stigma refers to a negative attitude formed by an individual’s negative cognition and negative emotional experience of depression. In contrast, perceived depression stigma is defined as the derogatory and rejection of depressed individuals by others (23). Studies have shown that the higher the level of depression stigma, the more negative the attitude and behavior of those seeking psychological help (24). Depression stigma may be an obstacle to people seeking mental health services.

This study aims to fill the existing gaps by assessing the prevalence of PHSI among Chinese medical students and identifying its associated factors. By doing so, it contributes to the understanding of how individual and academic pressures impact PHSI, and underscores the importance of addressing these factors to improve mental health support services on campus. The novelty of this study lies in its comprehensive approach to examining a range of factors affecting PHSI in a specific and under-researched population, thereby offering valuable insights for the design and implementation of targeted interventions.

## Methods

### Participants and sampling

This cross-sectional study was carried out in Hainan Province, China, from January 1, 2021, to May 31, 2021. Hainan Province is located on China’s southernmost tip. The land composition of Hainan province includes Hainan Island and Paracel Islands, the Spratly Islands, and the Zhongsha Islands. There are three medical schools in Hainan Province: Hainan Medical University, Hainan Health Management College, and Hainan Health Vocational College.

To ensure a comprehensive and representative sample, our study utilized a mixed sampling strategy, integrating both subjective quota sampling and objective random sampling techniques. The initial phase involved subjective quota sampling, where we aimed to include a wide array of medical disciplines. This was operationalized by randomly selecting five majors from among the three medical schools in Hainan Province, ensuring our sample reflected the diversity within medical education fields. The use of random selection in this phase served to minimize selection bias and provide an equitable representation of various medical disciplines. This random selection aimed to minimize bias in the choice of majors and to reflect the diversity of medical education in Hainan Province.

Due to the overlap between the majors selected by each school, 11 majors were finally selected, including preventive medicine, medical laboratory technology, clinical medicine, nursing, anesthesia, imaging, medical laboratory technology, pharmaceutical management and other medical majors.

Subsequently, to achieve a balanced representation of students across different years, one class was randomly selected from each grade of the chosen majors. This step combined the principles of random sampling with the need to manage the study’s practical aspects, such as accessibility and logistical feasibility. Students from the selected classes were then recruited as study participants, combining objective random sampling with subjective considerations to ensure a diverse and representative sample.

This survey was conducted with a class as a unit. The students of the selected classes were gathered in the classroom, and the uniformly trained investigators distributed and collected the questionnaires on the spot. Two team members verified the accuracy of the data and entered it into the EpiData 4.6.0.0 software.

To further ensure the ethical integrity of our research, specific attention was given to the protection of participant information and the secure handling of data. Prior to data collection, all participants were thoroughly informed about the study’s purpose, the confidentiality of their responses, and their right to withdraw from the study at any time without penalty. Informed consent forms, detailing the study’s objectives, potential risks, and benefits, were distributed to all participants. These forms were collected and stored securely, in compliance with data protection regulations, ensuring participants’ privacy and the confidentiality of their responses. Data collected through the questionnaires were anonymized and coded to prevent any identification of individual participants. Electronic data were encrypted and stored on secure, password-protected servers accessible only to the research team. These measures were implemented to safeguard the participants’ privacy and the integrity of the research data, reflecting our commitment to conducting our study in an ethically responsible manner.

Overall, 2,200 medical students were obtained from three schools, giving a response rate of 96.49%. Of the 2200 questionnaires collected, 38 (1.7%) were discarded due to logical errors or a large amount of missing information, leaving 2182 self-reported questionnaires for our analyses. The survey was piloted and refined with a convenience group of 60 medical students from Hainan Medical University.

### Measures

#### PHSI

We assess PHSI using one-item question, as suggested by Ebert (25). If they encounter psychological problems in the future, students rated their likelihood of seeking help from the campus counseling center or elsewhere, such as a mental health professional or doctor. There were four response options: definitely would go, probably would go, probably would not go, and definitely would not go.

The response options were then aggregated into two groups: those with PHSI (those who answered “definitely would go” or “probably would go”) and those without PHSI (those who answered “probably would not go” or “definitely would not go”) if they experience future psychological problems.

#### Barriers to utilization

If respondents indicated a reluctance to seek assistance for future psychological issues, opting against a definitive commitment to pursue help (“definitely would go”), further inquiry was conducted to elucidate the underlying reasons for such hesitance. Participants were presented with a series of potential deterrents, including: a lack of awareness regarding appropriate resources or professionals (“You do not know where to go or whom to see”); a preference for self-reliance in addressing the problem (“You would want to handle the problem on your own”); concerns about confidentiality and social stigma (“You are worried that others know that you have a psychological problem”); feelings of shame in discussing mental health issues with an unfamiliar individual (“You would be embarrassed to discuss mental health problems with a stranger”); skepticism about the efficacy of available treatments (“You feel that available treatments will not help”); a tendency to seek support from personal networks rather than professionals (“You would talk to relatives or friends instead”); and any other unspecified reasons (“Other reasons”). For each identified reason, respondents were asked to provide dichotomous responses (Yes/No), thereby enabling the capture of their perspectives on barriers to seeking psychological help.

#### Depression

The Patient Health Questionnaire (PHQ-9) is a widely used tool for screening for depression and assessing the severity of depression in the past two weeks (26). The items’ responses are provided on a 4-point Likert-type scale ranging from 0 (not at all) to 3 (almost daily). The item scores are summed to provide a total score (range: 0-27), with higher scores indicating higher levels of depression. Validated cut-off scores were used, with scores of 0–9 covering none to mild depression and 10–27 spanning moderate to severe depression.

The Chinese version of the PHQ-9 has been commonly used in China, with good reliability and validity (27). In the current study, Cronbach’s alpha was 0.90, indicating a high level of internal consistency.

#### Family function

The family function was assessed using the Family APGAR Index, developed by Smilkstein in 1987 (28). It consists of five items that assess satisfaction with family members’ social support in five domains: adaptability, partnership, growth, affection, and resolve. Responses are given on a three-point Likert scale ranging from 2 (often) to 0 (scarcely). The item scores are added together to produce a total score (range: 0-10), with higher scores indicating better family function. We used validated cut-off scores of 0 to 3 for severe family dysfunction, 4 to 6 for moderate family dysfunction, and 7 to 10 for good family functioning. The Family APGAR Index has been widely used in China, with good reliability and validity (29). In addition, the Family APGAR Index demonstrated high internal consistency in our study (Cronbach’s α = 0.86).

#### Personal depression stigma

Personal depression stigma was assessed using a 9-item sub-scale of the standardized Depression Stigma Scale (DSS-Personal scale) (30). Participants were polled on their attitudes toward depressed people (for example, “Depression is a sign of personal weakness”). Each item was graded on a 5-point Likert scale ranging from 0 (strongly disagree) to 4 (strongly agree) (strongly agree). The item scores are summed to provide a total score (range: 0–36), with higher scores indicating greater stigma. The scale is commonly used in different populations across cultures (21, 31). In this study, the Chinese version of the DSS-Personal stigma sub-scale was found to have moderate to high internal consistency (27), with a Cronbach’s alpha of 0.85.

We also collected data on socio-demographic characteristics: age, grade, sex (male/female), and the family structure (only child or non-only child). Other measures included self-rated academic performance level (far above average, slightly above average, average, slightly below average, or far below average) and self-rated dependence on mobile phones (light, moderate, or heavy).

### Statistical analyses

We used descriptive analysis to summarize respondents’characteristics. The frequency count with percentage was used for categorical data, while the mean with standard deviation (SD) or median and inter-quartile range was used for numerical variables (IQR). We used Chi-square and Rank-sum tests to compare PHSI by respondents’ characteristics. Furthermore, Chi-square test analyses were used to investigate the relationships between stigma item responses and PHSI. We applied logistic regression analyses to examine psycho-social correlates with PHSI among Chinese medical students with reporting odds ratios (ORs) and 95% confidence interval (CI). All comparisons were evaluated using two-tailed tests, with the significance level set at less than 0.05 (*P* < 0.05). All statistical analyses were performed using the Statistical Analysis System (SAS) 9.4 version for Windows (SAS Institute Inc., Cary, NC, USA).

## Results

**Table 1** summarizes the main characteristics of the students (n = 2182). The mean age of the students was 21.0 years (SD = 3.70), and 61.5% were female. In total, 32.9% of students were the only child in the family, and 21.8% rated their academic performance level as below average. Among the 2177 students who reported information on mobile phone use, 32.9% reported being heavily reliant on mobile phones. The median depression score of 2182 students were 5 (IQR=8), and the overall prevalence of depression was 16.4%. Although 72.0% of students reported having PHSI, only 13.6% answered that they “definitely would go” to seek help if they encountered future psychological problems. The barriers to utilization among the 1885 students who reported that they might not seek help if they encounter future psychological problems are primarily the desire to handle their problems on their own (59.8%), not knowing where to go for help (33.6%), preference to talk to friends or relatives rather than a mental health professional (28.7%), and embarrassment of discussing mental health problems with a stranger (21.9%).

**Table 1:** The main characteristics of respondents (n=2182).

Participant characteristics	N	%
**Age, Mean (SD)**	21.1 (3.7)	
Sex		
Male	840	38.5
Female	1342	61.5
Grade ^a^		
1	642	29.4
2	546	25.0
3	376	17.3
4	617	28.3
missing^a^	1	XX
Only child in the family(Missing=4)		
Yes	716	32.9
No	1462	67.1
Self-rated academic performance level		
Far above average/somewhat above average	590	27.0
Average	1116	51.2
Somewhat below average/far below average	476	21.8
Self-rated dependence on mobile phones(Missing=5)		
Light	315	14.5
Moderate	1145	52.6
Heavy	717	32.9
Family function		
Serious family dysfunction (APGAR index scores 0-3)	199	9.1
Moderate family dysfunction (APGAR index scores 4-6)	1425	65.3
Good family function (APGAR index scores 7-10)	558	25.6
Personal depression stigma level^b^		
4^th^ quintile (scores ≥18)	546	25.0
3^rd^ quintile (scores 15-18)	629	28.8
2^nd^quintile (scores 10-14)	444	20.3
1^st^ quintile (scores ≤10)	563	25.8
Depression		
No to minimal (PHQ-9 scores 0–9)	1825	83.6
Moderate to severe (PHQ-9 scores 10–27)	357	16.4
Professional help-seeking intention (PHSI)		
With PHSI	1572	72.0
With Non-PHSI	610	28.0

a Number of missing responses;

b Personal depression stigma is divided into four groups using the distribution’s quartiles.

For the associations between respondents’ characteristics and PHSI, **Table 2** shows a significant difference in PHSI by sex (*P* < 0.05), self-rated academic performance level (*P* = 0.01), self-rated dependence on mobile phones (*P* < 0.001), family function (*P* < 0.001), and depression stigma (*P* < 0.001).

**Table 2:** Professional help-seeking intention (PHSI) by the characteristics of respondents’ characteristics.

Participant characteristics	N	PHSI	
		N (%)	*P-value*
**Sex**			0.003
Male	838	566 (67.5)	
Female	1335	995 (74.5)	
**Grade**			0.205
1	642	465 (72.4)	
2	546	395 (72.3)	
3	376	281 (74.7)	
4	617	424 (68.7)	
**Only child in the family or not**			0.54
Yes	716	519 (72.3)	
No	1462	1045 (71.5)	
**Self-rated academic performance level**			0.010
Far above average/somewhat above average	590	425 (72.0)	
Average	1116	824 (73.8)	
Somewhat below average/far below average	476	317 (66.6)	
**Self-rated dependence on mobile phones**			<0.001
Light	315	253 (80.3)	
Moderate	1145	806 (70.4)	
Heavy	718	504 (70.2)	
**Family function**			<0.001
Serious family dysfunction	200	131 (65.5)	
Moderate family dysfunction	1423	986 (69.3)	
Good family function	560	449 (80.2)	
**Personal Depression stigma**			<0.001
4^th^ quintile(scores >=18)	546	352 (64.5)	
3^rd^ quintile(scores 15-18)	629	451 (71.7)	
2^nd^quintile (scores 10-14)	444	324 (73.0)	
1^st^ quintile (scores <=10)	563	439 (78.0)	

Regarding depression stigma, **Table 3** shows that students without PHSI felt more strongly than those with PHSI that depression is not an actual medical illness (24.1% VS 17.2%) and that people with depression are dangerous (33.3% VS 27.1%). Students without PHSI were also more likely to agree that if they were depressed, they would not tell anyone than students with PHSI (37.6% VS 22.5%). *P*< 0.01 is for all the preceding comparisons.

**Table 3:** The Stigma Responses^a^ and associations with PHSI.

Statements of the DSS-Personal scale^c^	PHSI^b^		*P*
	with PHSI	With non-PHSI	
People with depression could snap out of it if they wanted			0.114
disagree	1174(94.5)	411(92.4)	
agree	69(5.6)	34(7.6)	
Depression is a sign of personal weakness			0.233
disagree	680(58.2)	222(54.8)	
agree	488(41.8)	183(45.2)	
Depression is not a real medical illness			0.002
disagree	937(82.9)	302(75.9)	
agree	194(17.2)	96(24.1)	
People with depression are dangerous			0.018
disagree	830(72.9)	277(66.8)	
agree	309(27.1)	138(33.3)	
It’s best to avoid depressed people so you won’t have the same problems			0.065
disagree	1127(89.9)	406(86.8)	
agree	127(10.1)	62(13.3)	
People with depression are unpredictable 6			0.083
disagree	601(56.0)	202(50.9)	
agree	473(44.0)	195(49.1)	
If I had a depression I would not tell anyone			<0.0001
disagree	807(77.5)	255(64.4)	
agree	234(22.5)	141(37.6)	
I would not make friends with him if I knew he had been depressed			0.117
disagree	844(77.2)	294(73.3)	
agree	249(22.8)	107(26.7)	
I would not vote for a class cadre if I knew they had been depressed			0.464
disagree	594(55.2)	218(53.0)	
agree	483(44.9)	193(47.0)	

a Responses of “strongly agree” and “agree” were recorded as “agree”; “strongly disagree” and “disagree” were “disagree”; responses of “uncertain” were excluded from the analysis.

b PHSI, professional help-seeking intention.

c The DSS-Personal scale, the sub-scale of the standardized Depression Stigma Scale.

Our logistic regression analyses revealed that students who were male (OR = 1.5), had a high level of depression stigma (OR = 2.0), had serious family dysfunction (OR = 2.1), and reported heavy reliance on mobile phones (OR = 1.7) were less likely than their counterparts to have PHSI (**Table 4**).

**Table 4:** logistic regression analysis for factors associated with depression and Non-PHSI.

Variables	OR (95% CIs)
Gender	
Male	1.5 (1.2-1.9)***
Female	1 (Ref)
Grade	
1	1 (Ref)
2	1.0 (0.8-1.3)
3	0.9 (0.6-1.2)
4	1.2 (0.9-1.6)
Only child in the family or not	
Yes	1 (Ref)
No	1.0 (0.8-1.3)
Self-rated academic performance level	
Far above average/somewhat above average	1 (Ref)
Average	0.9 (0.7-1.2)
Somewhat below average/far below average	1.2 (0.9-1.6)
Self-rated dependence on mobile phones	
Light	1 (Ref)
Moderate	1.6 (1.1-2.5)**
Heavy	1.7 (1.2-2.5)**
Family function	
Serious family dysfunction	2.1 (1.3-3.2)***
Moderate family dysfunction	1.6 (1.2-2.2)***
Good family function	1 (Ref)
Depression Stigma^c^	
4^th^ quintile(scores >=18)	2.0 (1.5-2.7)***
3^rd^ quintile(scores 15-18)	1.5 (1.1-2.0)*
2^nd^quintile (scores 10-14)	1.4 (1.0-1.9)
1^st^ quintile (scores <=10)	1 (Ref)
Depression	
No to minimal (PHQ-9 scores 0–9)	1 (Ref)
Moderate to severe (PHQ-9 scores 10–27)	1.5 (1.1-1.9)**

a OR, odds ratio; 95% CI, 95% confidence interval; # Adjusted for all other variables listed in the table.

b Non-PHSI, non-professional help-seeking intention.

c According to the depression stigma quartiles, the data is divided into four groups;1^st^ quartile means that twenty-five percent of sample observations are less than or equal to the value of the first quartile.

*P < 0.05, **P < 0.01, ***P < 0.001.

## Discussion

The pressing need to address mental health among medical students stems from the unique pressures and challenges they face, including intense academic demands and exposure to patient suffering. This study, being one of the first to explore PHSI within this population in China using the Mindsponge Theory, offers novel insights into the complex interplay of personal, familial, and societal factors influencing their mental health. Medical students are future doctors. Therefore, reliable estimates of the PHSI among them are critical, especially given recent studies indicating a high prevalence of depression among resident physicians (32). In our study, 16.4% of students exhibited moderate to severe depression symptoms. This finding suggests that while a significant minority of medical students are affected, depression does not predominate across the entire student population. Identifying characteristics of high-risk individuals becomes a crucial aspect of our analysis.

Depression was reported to be prevalent among medical students in America (17.0%) (33), Mexico (16.2%) (34), and New Zealand (16.9%) (35), all of which were comparable with findings. It is worth mentioning that the same depression screening instrument (PHQ-9) was used in the above-mentioned studies, including ours.

Moreover, although our study showed that 72.0% of the students with PHSI, only 13.6% “definitely would go” to seek help if they encountered future psychological problems. This willingness demonstrated by the 13.6% underscores a strength in mental health literacy and a readiness to overcome stigma, a vital trait that could be leveraged in designing interventions aimed at increasing PHSI among their peers.

Although most colleges and universities have established relevant institutions that provide mental health services, this study found that, while nearly three-quarters of medical students reported having PHSI, 28% had no intention of seeking such professional help once they graduated; medical students may be a risk group with insufficient utilization of mental health services. The reluctance of these 28% of students highlights areas for improvement, especially in addressing personal and perceived barriers to seeking help. Understanding these weaknesses is pivotal for tailoring recommendations that could facilitate a more supportive environment for help-seeking behavior. Concerning utilization barriers, our study found that the top perceived utilization barrier among medical students was “the desire to handle their problems on their own.” This barrier among our medical students is consistent with previous research, which reported that the individuals who believed they could solve their mental health problems were reluctant to admit their problems and had negative attitudes toward seeking professional help (36).

Our findings also show that male college students had lower PHSI than female students (68.0% VS 74.6%). The previous studies from Turkey (37) and Norway reported similar results (18). Self-disclosure may be essential, and females are more willing to disclose themselves than males from the perspective of social psychology (16). Women are more likely to seek emotional support and talk to others once faced with a stressful event or negative emotions. In traditional stereotypes, men are virtuous as a “tool of silence” and should not experience unfavorable emotional fluctuations. “A man’s tears do not flick easily,” according to an old Chinese proverb. Men are generally thought to be more robust than women. Men were likely to be more stressed when seeking professional psychological help under the influence of this type of gender role conflict, and they are more likely to have a negative attitude and a lower PHSI (38). Considering these beliefs, it is necessary to develop gender-sensitive help-seeking interventions for male students based on evidence-based practice in the future. In the present study, family dysfunction was significantly associated with lower PHSI. This finding is in line with a study conducted in Turkey (37). Individuals and families in China are inextricably linked in the context of collectivist culture, and family is the most important social support network for Chinese college students. Good family functionality allows individuals to feel loved, cared for, and respected within the family, resulting in a positive emotional experience in social life. It also acts as a powerful buffer against social pressure (39). Compared with students with low-income family functionality, students with good family functions may interact with their families more often and establish trusting relationships. This interaction may include conversation and exchange of ideas (40). Therefore, if family members have positive attitudes toward seeking professional psychological help, students may be influenced by them and form more positive attitudes.

Furthermore, this study found that self-reported mobile phone addiction is associated with lower PHSI. Smartphones are now an essential part of daily life, bringing convenience to everyone’s lives. However, college students excessive use of mobile phones can have negative consequences, such as disrupting sleep and schoolwork, as well as stress and other psychological issues (41–43). In addition, college students suffering from depression may use smartphones to cope with negative emotions or as an “exit” to relieve stress (43). They are obsessed with mobile technology and are unwilling to seek professional psychological help, creating a vicious circle of poor mental health. Therefore, college students should be informed about the potential risks associated with excessive smartphone use and encouraged to use smartphones responsibly. In light of digital technology’s growing role, a cross-country study by Crocamo suggests that digital platforms, like apps and social media, could effectively disseminate mental health support and information (44). These tools offer anonymity and wide availability, key for students hesitant to seek help due to stigma. Integrating such digital solutions could complement existing mental health services, potentially enhancing help-seeking among Chinese medical students.

Finally, it is worth noting that stigma is strongly linked to depression and lower PHSI. Public stigma is the negative impression of society on the help-seeking group (45). Many people with depression refuse psychological help or hide their condition to avoid being labeled (46). When people with mental illness internalize public stigma, it can lead to personal stigma and lower levels of self-esteem, believing themselves to be flawed and pathetic. We also discovered that medical students regard depression as an unpredictable and personal weakness. Physical illnesses are easier to receive sympathy and acceptance in traditional Chinese culture. However, the Chinese tend to define mental illness as a personal weakness and relate it to moral character. The deep-rooted cultural stigma around mental health in China, compounded by Confucian ideals of self-reliance and stoicism, exacerbates the challenges faced by those needing psychological help. The reluctance to seek such help is not merely a personal choice but is heavily influenced by the broader societal expectation to uphold familial honor and personal strength. It is crucial, therefore, for interventions in China to address these cultural barriers head-on, fostering an environment where seeking mental health support is not seen as a detriment to one’s character or family reputation. Young college students who conflict with the accepted prerequisites of interpersonal dependence and independence, intimacy and avoidance of such are eager to advance in the group and gain understanding and respect from others (47). As a result of their fear of discrimination and rejection by others in interpersonal communication, they choose to hide and avoid psychological help.

Mindsponge Theory (MT) elucidates how individuals selectively absorb, process, and either adopt or reject cultural and informational inputs based on their cognitive and environmental contexts (48). This framework is particularly relevant in understanding the dynamics of professional help-seeking intentions (PHSI) among Chinese medical students, as it highlights the role of cognitive biases and societal norms in shaping attitudes toward mental health. The theory suggests that students’ willingness to seek help is influenced by their ‘absorption’ or ‘rejection’ of attitudes towards mental health, which is mediated by factors such as gender differences, family functionality, mobile phone addiction, and cultural stigma. For instance, cultural stigma and gender norms might lead some students to ‘reject’ the idea of seeking help, adhering instead to societal expectations of strength and self-reliance. Conversely, students from supportive family backgrounds may be more inclined to ‘absorb’ positive views on mental health care, thus being more likely to seek help when needed. By applying Mindsponge Theory, we can better appreciate the importance of addressing both cognitive biases and societal influences to enhance PHSI among this demographic. To combat the identified barriers to PHSI, we recommend the implementation of comprehensive mental health education programs that promote awareness and destigmatization, enhanced access to supportive services, and the integration of digital health solutions. Collaborative efforts involving educational institutions, healthcare providers, and policymakers are essential to create a supportive environment conducive to mental health help-seeking.

There are several limitations to our study. First, cross-sectional data limits our ability to establish causal relationships between study variables, so future research using a cohort design is preferred. Additionally, the use of self-reported questionnaires introduces biases such as social desirability and recall inaccuracies, which might affect the objectivity and reliability of the reported data. Third, our study’s geographical scope was confined to medical students in a specific southern province, limiting the generalizability of our findings to the broader population of medical students across China.

## Conclusion

This study sheds light on professional help-seeking intention (PHSI) among Chinese medical students, revealing that a majority express willingness to seek psychological help. However, key factors such as gender, depression stigma, family dysfunction, and mobile phone dependence significantly influence their PHSI. Particularly, male students and those experiencing higher levels of stigma or family dysfunction show lower PHSI. These insights underscore the importance of addressing both personal and environmental barriers to enhance PHSI in future healthcare professionals.

## Data availability statement

The original contributions presented in the study are included in the article/supplementary material. Further inquiries can be directed to the corresponding authors.

## Ethics statement

This study was conducted in accordance with the principles outlined in the Declaration of Helsinki and was approved by the Human Research Ethics Committee, Hainan Medical University, Haikou, China (HYLL-2022-210). After the study procedures were explained to the participants, written informed consent was obtained from them in accordance with the Declaration of Helsinki.

## Author contributions

LQ: Funding acquisition, Writing – original draft, Writing – review & editing. KW: Data curation, Formal analysis, Writing – review & editing. YL: Investigation, Software, Writing – review & editing. JD: Writing – review & editing. HL: Formal analysis, Validation, Writing – review & editing. JM: Data curation, Investigation, Writing – review & editing.
